# Design of a Blockchain-Enabled Traceability System for *Pleurotus ostreatus* Supply Chains

**DOI:** 10.3390/foods14223959

**Published:** 2025-11-19

**Authors:** Hongyan Guo, Wei Xu, Mingxia Lin, Xingguo Zhang, Pingzeng Liu

**Affiliations:** 1 School of Information Science and Engineering, Shandong Agricultural University, Tai’an 271018, China; ghy11042025@163.com (H.G.); xuwei202425@163.com (W.X.); linmingxia666@163.com (M.L.); xingguo_zhang@163.com (X.Z.); 2 Key Laboratory of Huang-Huai-Hai Smart Agricultural Technology, Ministry of Agriculture and Rural Affairs, Tai’an 271018, China; 3 Agricultural Big-Data Research Center, Shandong Agricultural University, Tai’an 271018, China

**Keywords:** blockchain, IoT in agriculture, *Pleurotus ostreatus*, agricultural product safety, data fusion

## Abstract

*Pleurotus ostreatus* is valued for its nutritional, medicinal, economic, and ecological benefits and is widely used in the food, pharmaceutical, and environmental protection industries. *Pleurotus ostreatus*, as a highly perishable edible fungus, faces significant challenges in supply chain quality control and food safety due to its short shelf life. As consumer demand for food freshness and full traceability increases, there is an urgent need to establish a reliable traceability system that enables real-time monitoring, spoilage prevention, and quality assurance. This study focuses on the *Pleurotus ostreatus* supply chain and designs and implements a multi-role flexible traceability system that integrates blockchain and the Internet of Things. The system collects key production and storage environment parameters in real time through sensor networks and enhances data accuracy and robustness using an improved adaptive weighted fusion algorithm, enabling precise monitoring of the growth environment and quality risks. The system adopts a “link-chain” mapping mechanism for multi-chain storage and dynamic reorganization of business processes. It incorporates attribute-based encryption strategies and smart contracts to support tiered data access and secure sharing among multiple parties. Key information is stored on the blockchain to prevent tampering, while auxiliary data is stored in off-chain databases and the Interplanetary File System to ensure efficient and verifiable data queries. Deployed at Shandong Qihe Ecological Agriculture Co., Ltd., No. 517, Xilou Village, Kunlun Town, Zichuan District, 255000, Zibo City, Shandong Province, China, the system covers 12 cultivation units and 60 sensor nodes, recording over 50,000 traceable data points. Experimental results demonstrate that the system outperforms baseline methods in query latency, data consistency, and environmental monitoring accuracy. The improved fusion algorithm reduced the total variance of environmental data by 20%. In practical application, the system reduced the spoilage rate of *Pleurotus ostreatus* by approximately 12.3% and increased the quality inspection pass rate by approximately 15.4%, significantly enhancing the supply chain’s quality control and food safety capabilities. The results show that the framework is feasible and scalable in terms of information credibility and operational efficiency and significantly improves food quality and safety monitoring throughout the production, storage, and distribution of *Pleurotus ostreatus*. This study provides a viable technological path for spoilage prevention, quality tracking, and digital food safety supervision, offering valuable insights for both food science research and practical applications.

## 1. Introduction

### 1.1. Background

China is the world’s largest producer and exporter of edible mushrooms [1]. Edible mushrooms hold a significant position in Chinese agriculture, ranking as the fifth-largest agricultural plantation sector [2]. Valued for their medicinal properties, nutritional content, and health benefits, edible mushrooms have experienced rapid growth in international markets [3]. They have also become new focal points and growth drivers for rural economic development, indicating strong development potential [4].

However, studies have shown that edible fungi possess a natural ability to bioaccumulate heavy metals [5]. Contamination of raw materials and non-standardized practices across cultivation, storage, processing, and logistics stages in the edible mushroom supply chain have led to concerns such as excessive pesticide residues and heavy metal accumulation, exacerbating food safety risks.

As one of the most widely cultivated mushroom species in China, *Pleurotus ostreatus* accounts for 16.51% of the country’s total edible mushroom production [6]. Its cultivation is particularly vulnerable to soil contamination, increasing the likelihood of heavy metal bioaccumulation. Therefore, establishing an effective product quality and safety traceability system is essential for improving the management and oversight of *Pleurotus ostreatus* quality and safety.

Currently, the frequent occurrence of food contamination incidents, weak regulatory frameworks, and high risks of data tampering pose serious threats to public health and consumer trust. These challenges are particularly acute for *Pleurotus ostreatus* (oyster mushroom), a highly perishable and sensitive agricultural product. Its short shelf life, dependence on cold-chain logistics, and potential for heavy metal accumulation further exacerbate quality and safety risks [7]. Against this backdrop, there is an urgent need to establish a trustworthy traceability system that involves multiple stakeholders and spans the entire supply chain, thereby ensuring comprehensive quality and safety assurance from production to consumption.

### 1.2. Related Work and Motivation

Accurate collection of product safety information forms the foundation of traceability. This involves technologies that utilize information systems for the automatic identification, collection, and input of data into computer systems. Driven by advances in information technology and increasing government emphasis on food safety traceability, researchers have continuously innovated data collection methods. Widely adopted technologies currently include sensor networks [8], Electronic Identification (EID) [9], Radio Frequency Identification (RFID) [10], two-dimensional (2D) barcode technology [11], Global Positioning System (GPS) [12], and image recognition [13].

As the primary growth environment for *Pleurotus ostreatus*, accurate environmental data collection within mushroom rooms directly affects the quality and safety control of the mushrooms. Studies indicate that the growth environment significantly influences the bioaccumulation of heavy metals in edible fungi. Specifically, mushrooms cultivated under optimal conditions exhibit substantially lower heavy metal accumulation compared to those grown in suboptimal environments. In the field of *Pleurotus ostreatus* production, IoT technologies have been widely applied to environmental monitoring and intelligent control, enabling more refined management of the cultivation process [14,15,16]. At the same time, RFID systems have shown considerable potential in storage management and logistics tracking; for instance, optimized deployment strategies for different storage structures can improve both data collection efficiency and traceability accuracy [17,18]. However, relying solely on centralized architectures such as IoT and RFID still faces limitations, including vulnerability to data tampering and difficulties in establishing mutual trust among stakeholders [19].

Traditional centralized data storage methods suffer from vulnerabilities such as data tampering and forgery. In recent years, blockchain technology has been adopted across various agricultural supply chains [20,21,22,23,24,25], enabling tamper-proof information storage and end-to-end traceability. Nonetheless, existing blockchain traceability systems still face challenges regarding applicability and meeting the diverse needs of different traceability stakeholders. Existing studies have verified from multiple perspectives that blockchain and related technologies can enhance supply chain management [26,27,28,29,30]. However, most of these works focus on general agricultural or industrial products, with insufficient attention given to edible fungi, which are highly perishable and extremely sensitive to cold-chain and environmental conditions. To date, there has been no systematic solution that simultaneously addresses quality risk monitoring, trustworthy recording of process data, and rapid product recall in this context.

Although existing studies have achieved substantial progress in integrating IoT, blockchain, and supply chain management, there remains a lack of in-depth research on *Pleurotus ostreatus*, a highly perishable product with strong dependence on environmental conditions. Current systems largely rely on IoT-based environmental monitoring but lack trustworthy data storage and flexible traceability mechanisms, making it difficult to address the differentiated needs across various supply chain stages. In addition, traceability information from multiple stakeholders is often stored within a single blockchain ledger, which limits the system’s ability to meet heterogeneous requirements [31].

To overcome these limitations, it is essential to build a trustworthy traceability framework capable of simultaneously ensuring environmental quality monitoring, spoilage risk control, and food safety assurance. Therefore, this study proposes a flexible traceability system for the *P. ostreatus* supply chain that combines real-time IoT sensing with blockchain-based trustworthy storage. Across cultivation, storage, and cold-chain transportation stages, the system records and traces key food safety indicators—including temperature, humidity, CO_2_ concentration, batch inspection results, and recall responses—in a secure and verifiable manner. The primary goals are to reduce spoilage risk, improve the accuracy and efficiency of incident localization and recall, and provide regulatory agencies with auditable evidence. This framework addresses existing research gaps and offers a novel digital solution for food preservation, safety assurance, and quality control.

### 1.3. Our Contributions

To address the above challenges, this study proposes a blockchain- and IoT-enabled flexible traceability framework tailored for *Pleurotus ostreatus* cultivation. The major contributions of this work are summarized as follows:

1. We design a novel “process-to-chain” mapping mechanism that decouples the production workflow into seven modular sub-chains. This mechanism allows for the dynamic reorganization of traceability paths based on different production scenarios and stakeholder needs, enabling flexible supervision and detailed traceability of the supply chain for highly perishable edible fungi.
2. A three-layer access control model—comprising public, private, and regulatory domains—is established. By integrating Ciphertext-Policy Attribute-Based Encryption (CP-ABE) with customized smart contracts, the system enables secure and precise data sharing among producers, regulators, and consumers, thereby supporting the trustworthy circulation of *Pleurotus ostreatus* safety information.
3. A hybrid management approach combining blockchain and distributed storage has been adopted to ensure tamper-proof recording and verifiable querying of key quality data for *Pleurotus ostreatus*, while also enhancing the storage efficiency and privacy protection of environmental monitoring data.
4. A LoRa-based wireless sensor network has been established, and an improved adaptive weighted fusion algorithm has been introduced to effectively enhance the consistency and robustness of multi-point data collection. Experiments show that, compared to traditional averaging methods, the total variance is reduced by 20%, significantly improving the accuracy of environmental data and the sensitivity of spoilage risk monitoring.
5. The system has been deployed and is operational at Shandong Qihe Ecological Agriculture Co., Ltd., recording over 10,000 traceable events. The results show that, after the system’s implementation, the spoilage rate of *Pleurotus ostreatus* decreased by approximately 12.3%, and the quality inspection pass rate increased by about 15.4%. The system also outperforms traditional solutions in query latency, data consistency, and scalability. This demonstrates its feasibility and practical value in the quality control of edible fungi and food safety assurance.

### 1.4. Organization

Section 2 analyzes the quality and safety risks across the entire *Pleurotus ostreatus* industry chain and, in response, proposes a flexible and trustworthy traceability framework based on a multi-layer blockchain architecture and dynamic granularity adjustment. This framework ensures secure data management, privacy protection, and efficient traceability throughout the production process. Section 3 presents the system design, including a multi-chain dynamic tracking mechanism, a traceability code encoding scheme, and a rapid traceability module. An IoT-driven, multi-agent chain storage architecture is constructed to support secure and efficient data acquisition, storage, and access across diverse roles and application scenarios. Section 4 describes the implementation of the proposed traceability system using the Hyperledger Fabric platform. It supports end-to-end data collection and on-chain recording throughout the oyster mushroom supply chain, enabling segmented chain storage and dynamic workflow scheduling. These features significantly improve system security, transparency, and flexibility. Section 5 reports experimental comparisons, demonstrating that the MFT-Chain model outperforms conventional single-chain architectures in both query efficiency and system throughput, thus offering enhanced scalability and overall performance. Section 6 and Section 7 provide a comprehensive discussion of the system and summarize the main research findings and future development directions, respectively.

## 2. Design of a Quality and Safety Traceability Solution for *Pleurotus ostreatus*

### 2.1. Related Technologies

#### 2.1.1. Blockchain

**Figure 1:**
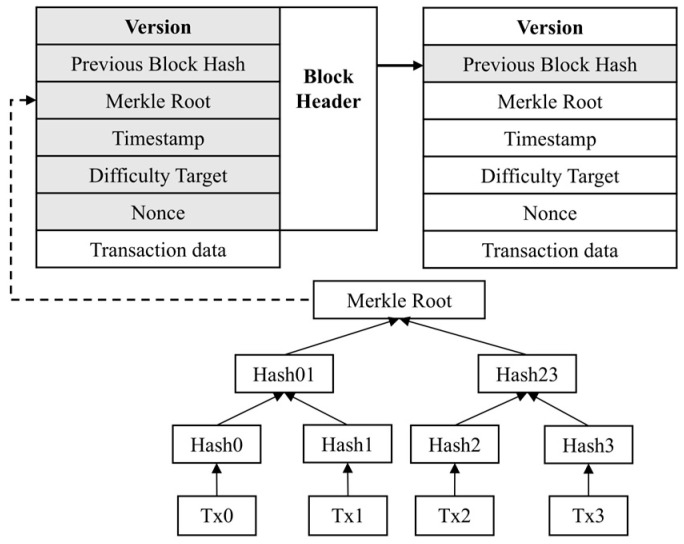
Blockchain structure diagram. (Arrows indicate the flow of data from the block header to the Merkle root and the connection between hash nodes and transaction data).

Blockchain is a decentralized distributed ledger technology characterized by data immutability, decentralization, transparency, and traceability [32]. As shown in Figure 1, the block body stores transaction data and constructs a Merkle tree through hashing, with the root hash recorded in the block header to ensure data integrity and consistency [33]. Data updates rely on consensus mechanisms such as Proof of Work (PoW), Proof of Stake (PoS), and Practical Byzantine Fault Tolerance (PBFT). A transaction is confirmed and added to the blockchain only when the majority of nodes reach consensus [34]. Since data cannot be altered once recorded, blockchain offers significant advantages in security and data trustworthiness [35].

#### 2.1.2. Internet of Things

The Internet of Things (IoT), a key technology in modern information systems, typically consists of three layers: perception, network, and application [36].

- The perception layer gathers real-world data through sensors, RFID, and other devices, providing precise inputs [37].
- The network layer transmits data and connects sensing devices to higher-level applications. LoRa (Long Range) technology is commonly used here for long-distance, low-power communication due to its Chirp Spread Spectrum modulation, which offers greater sensitivity than traditional FSK methods [38,39].
- The application layer processes and analyzes the sensory data, integrating it into industry-specific scenarios to support intelligent decision-making, visualization, and user services.

### 2.2. Comprehensive Analysis of the Pleurotus ostreatus Industry Chain

**Figure 2:**
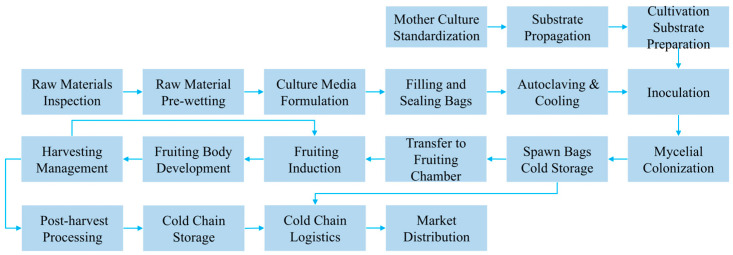
*Pleurotus ostreatus* production flow chart.

The *Pleurotus ostreatus* traceability system provides full-process tracking for the entire production and supply chain. By recording and transmitting key information, it enables accurate tracing and tracking of the origin and destination of *Pleurotus ostreatus*, supporting comprehensive monitoring and management of the production process. The supply chain is analyzed in detail, covering all stages of production, from raw material processing, fungus stick preparation, strain cultivation, and fruiting body growth to the sale of mature mushrooms, as shown in Figure 2.

Key quality and safety issues in the *Pleurotus ostreatus* supply chain not only include a lack of transparency in information management but also involve food safety risks such as microbial contamination, heavy metal accumulation, and the loss of temperature and humidity control in the cold chain. To effectively identify critical quality risks and develop targeted prevention strategies, Table 1 summarizes the risk points of quality and safety issues commonly encountered at each stage of the *Pleurotus ostreatus* supply chain based on the company’s actual conditions. This provides a foundational application scenario for the blockchain-based intelligent regulatory system. These issues can lead to fluctuations in product quality and may trigger food safety incidents such as heavy metal exceedances, cross-contamination, and false labeling.

### 2.3. System Architecture

**Figure 3:**
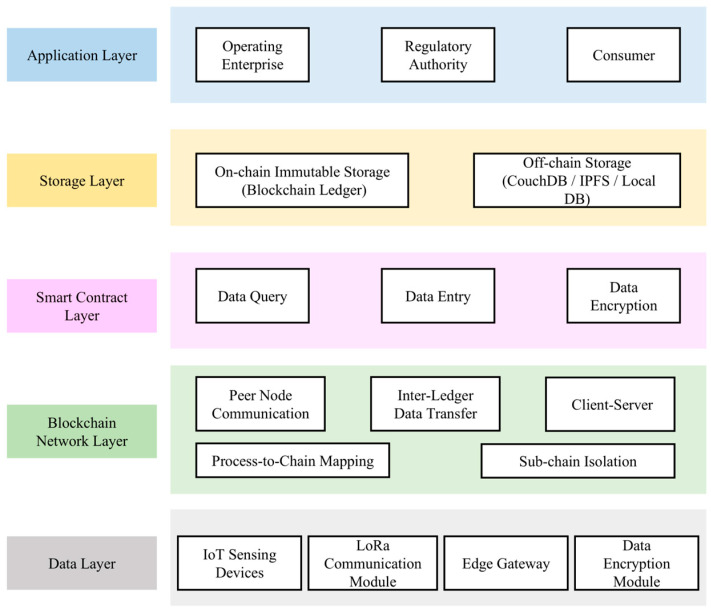
System architecture diagram of the *Pleurotus ostreatus* traceability system.

To effectively address the challenges of managing multi-source heterogeneous data and enabling multi-role collaborative traceability, this study proposes a five-layer traceability system architecture. The architecture consists of the data layer, blockchain network layer, smart contract layer, storage layer, and application layer, as depicted in Figure 3.

#### 2.3.1. Data Layer

This layer focuses on ensuring the authenticity and integrity of the data, forming the core foundation of traceability reliability. It is responsible for real-time collection of environmental parameters (such as temperature, humidity, CO_2_ concentration, etc.) through LoRa wireless sensor networks and edge computing nodes. To ensure data quality, this layer integrates anomaly detection and weighted fusion algorithms to suppress noise and provide reliable calibration of the collected data. It can reflect changes in the mushroom house’s ecological environment in real time, predict microbial growth risks, and support early quality intervention. These real-time monitoring data not only provide technical support but also serve as the first line of defense for quality and safety monitoring. They enable the timely detection of abnormal environmental conditions, reduce contamination or spoilage risks, and lay the groundwork for subsequent food safety assurance.

#### 2.3.2. Blockchain Network Layer

This layer records key events in the *Pleurotus ostreatus* supply chain on the blockchain in an immutable manner. Using an “on-chain hash pointer + off-chain raw data” approach, it stores indexes for large-scale data such as sensor curves and inspection reports. This ensures data authenticity and verifiability while preventing regulatory blind spots caused by missing or tampered information. Different stakeholders access the required information through hierarchical permissions: consumers view transparent compliance results, enterprises conduct end-to-end quality monitoring and management optimization, and regulators perform precise inspections based on a complete evidence chain. The mechanism not only safeguards the integrity and trustworthiness of critical data but also supports early risk identification, accurate product localization, and efficient recalls. By enabling intelligent responses to non-compliant indicators, the system enhances the handling efficiency of food safety incidents, reduces contamination spread, and mitigates health risks for consumers.

#### 2.3.3. Smart Contract Layer

As the system’s core logic layer, smart contracts manage data validation, process automation, and enforcement of operational rules across all stages of production and distribution. Built on a multi-chain architecture, the contracts execute pre-defined rules to automate data verification and submission tasks in production, processing, and logistics. Especially for *Pleurotus ostreatus*, which is highly sensitive to environmental conditions, the system can automatically trigger early-warning rules for temperature or humidity exceedance and batch anomalies, thereby establishing a “detect-and-respond” closed loop for food safety assurance.

#### 2.3.4. Storage Layer

To achieve both efficient data handling and secure storage, this layer adopts a hybrid on-chain/off-chain storage architecture. The blockchain stores only critical metadata or hashed references, while large-scale auxiliary data is encrypted and stored off-chain. Specifically, the InterPlanetary File System (IPFS) manages encrypted raw files, and a traditional relational database handles high-volume auxiliary information such as environmental logs and logistics records. While ensuring data reliability, this design also provides strong support for subsequent quality accountability, incident analysis, and cross-departmental joint supervision. It promotes a shift in *Pleurotus ostreatus* quality and safety regulation from post-incident investigation to proactive early warning.

#### 2.3.5. Application Layer

This layer provides tailored services for three types of users: consumers, enterprises, and regulators. Consumers can access detailed product information—such as origin, harvest time, and inspection reports—via QR code scanning or traceability code input. Enterprises can monitor the full lifecycle of products to strengthen quality control, optimize operations, and reduce waste. Regulatory agencies can leverage real-time traceability data for precise, dynamic oversight of the entire supply chain. The system supports both web and mobile interfaces, featuring intuitive user interaction, data visualization, and export capabilities to enhance accessibility and regulatory efficiency.

### 2.4. Traceability Granularity

Traceability granularity refers to the smallest unit of information in a traceability system [40]. Its size directly affects system accuracy and efficiency [41]. In flexible traceability design, granularity must balance accuracy, efficiency, and management costs [42]. Based on an analysis of critical issues across the *Pleurotus ostreatus* supply chain, this study defines granularity for seven stages: fungus stick production, inoculation, mushroom cultivation, storage, processing, transportation, and sales. These correspond to raw material batch, inoculation batch, harvest batch, storage batch, processing batch, transportation batch, and individual traceability code, respectively, as shown in Table 2.

**Figure 4:**
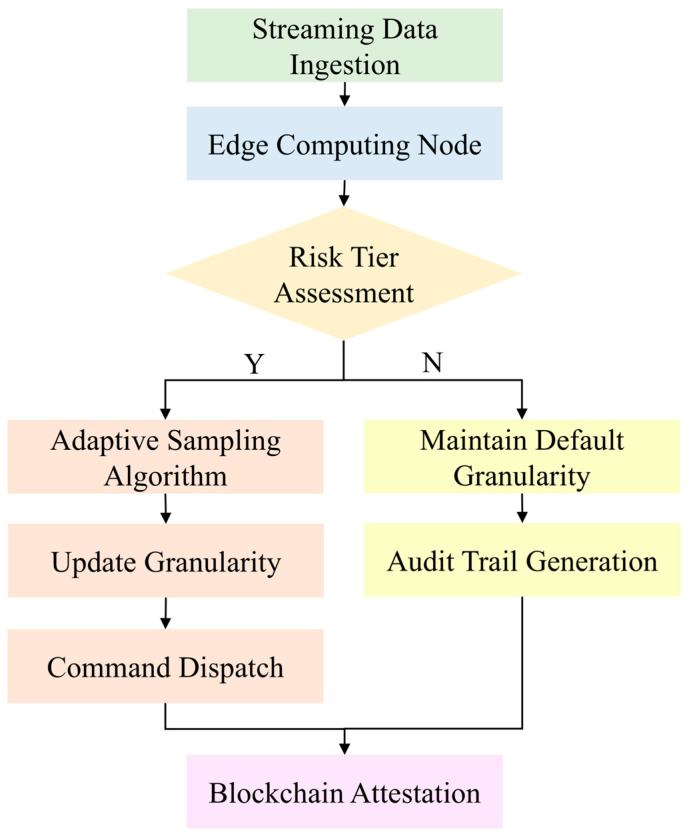
Dynamic traceability granularity adjustment mechanism.

Although this granularity division satisfies basic traceability needs, sudden quality incidents during production require dynamic granularity adjustments. Therefore, a dynamic adjustment mechanism is proposed, illustrated in Figure 4, which uses preset thresholds and smart contracts to execute granularity conversion algorithms for flexible adaptation. For example, in the event of contamination, inspection anomalies, or consumer complaints, the system can immediately adjust traceability granularity to quickly pinpoint the problematic batch or individual item. This enables the shortest path from “issue detection” to “issue resolution,” significantly enhancing food safety responsiveness and recall efficiency while minimizing the risk of secondary spread. The mechanism not only strengthens the system’s capacity for risk perception and control in emergency situations but also reduces the likelihood of spoilage or contamination diffusion. At the same time, it ensures precise recalls with clear accountability and reduces unnecessary waste caused by discarding entire batches, thereby achieving a balance between food safety and sustainable development.

## 3. Pleurotus ostreatus Product Quality and Safety Traceability System

### 3.1. Blockchain-Based Flexible Traceability Model for Pleurotus ostreatus

**Figure 5:**
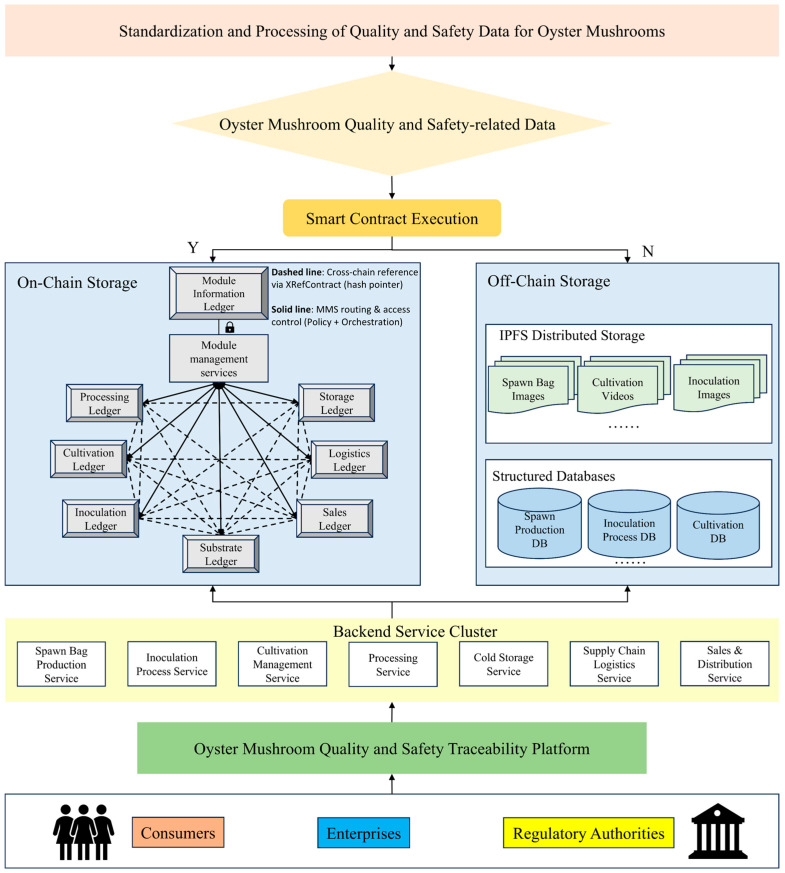
Blockchain-based flexible traceability model for *Pleurotus ostreatus*.

In this study, we propose a sub-chain network flexible traceability model built on a blockchain architecture, termed MFT-Chain, which is designed to enhance traceability flexibility and trustworthiness across the supply chain. This model enhances traceability flexibility and trustworthiness across the supply chain through a sub-chain network framework based on a “stage-to-ledger” mapping strategy, where each supply chain stage is designed as a modular, pluggable unit. These independent stage modules are coordinated by a centralized Module Management Service (MMS) that enables dynamic workflow composition and flexible reconfiguration of the supply chain. The system adopts a “one stage–one ledger” architecture, where each sub-chain independently handles data uploading, transaction validation, and traceability processing for its respective stage. As illustrated in Figure 5, this architecture ensures logical isolation between stages while supporting full-process traceability with high modularity and scalability. The coordination between sub-chains is achieved through smart contracts, which are responsible for verifying data integrity and ensuring consistency across different sub-chains. Cross-chain data references are implemented using a hash pointer mechanism, where each transaction record in a sub-chain generates a hash value that is validated and referenced through smart contracts, ensuring that data remains tamper-proof during its transmission across multiple sub-chains. Inter-module communication is decoupled via service-based orchestration, enhancing flexibility and adaptability across different operational scenarios.

In this framework, the main stages of the *Pleurotus ostreatus* supply chain—including inoculation, cultivation, storage, processing, transportation, and sales—are encapsulated into functionally autonomous modules. Each module integrates back-end services and a blockchain peer node with write permissions for the corresponding sub-ledger. These modules operate independently, executing on-chain recordkeeping, transaction verification, and traceability queries. The MMS is responsible for dynamically scheduling the execution order of each sub-chain, ensuring coordination between the stages and smooth process flow. Specifically, the MMS determines when to start or pause a sub-chain based on production requirements, event triggers, or pre-set rules. For instance, after the sub-chain for the Inoculation stage completes execution and confirms data integrity, the MMS automatically triggers the execution of the Cultivation sub-chain. In addition to scheduling, the MMS also takes on the task of coordinating data across sub-chains, ensuring data consistency and smooth flow between stages. Particularly, the MMS manages data flow and operational permissions between sub-chains through routing and access control, ensuring effective management of data transfer and permissions. This guarantees the continuity and integrity of the entire traceability system. The MMS serves as the system’s control hub, responsible for coordinating inter-module interactions, orchestrating workflow transitions, and facilitating real-time visualization for regulators and end users.

By leveraging blockchain’s distributed architecture, this flexible traceability system overcomes the rigidity of traditional linear traceability models. It not only enhances the system’s adaptability to unexpected quality issues but also effectively reduces the risk of spoilage caused by cold-chain failures or contamination at specific stages. By precisely identifying problematic links and rapidly isolating defective products, the system safeguards the baseline of food safety while minimizing unnecessary waste, thereby providing strong support for both food safety assurance and sustainable development.

### 3.2. Flexible Traceability Mechanism

#### 3.2.1. Dynamic Tracing Mechanism

To support diverse production workflows and evolving quality supervision needs, the system integrates a reconfigurable dynamic tracking mechanism. Following the software engineering principle of “high cohesion, low coupling,” the overall architecture is modularized into function-specific components with standardized interfaces. These modules can be flexibly assembled and reused based on real-world business scenarios, allowing seamless transitions and iterative execution of processes. For example, in a workflow such as “substrate preparation → inoculation → cultivation → storage → transportation → processing → storage → transportation → sales,” the storage and transportation modules may be invoked multiple times. This design ensures consistent data acquisition and process management even when stages are repeated, enabling reliable and traceable operations across complex production cycles.

To standardize data exchange between modules, the system introduces a unified data identification framework, which explicitly defines the data types, formats, and traceability fields for each supply chain stage. This framework forms the basis for interoperable module interaction and enhances the scalability and maintainability of the flexible traceability system under variable workflow conditions. The corresponding pseudocode logic for the dynamic tracking mechanism is presented in Algorithm 1.
**Algorithm 1**: Pseudocode for the Reconfigurable Dynamic Tracing Mechanism.

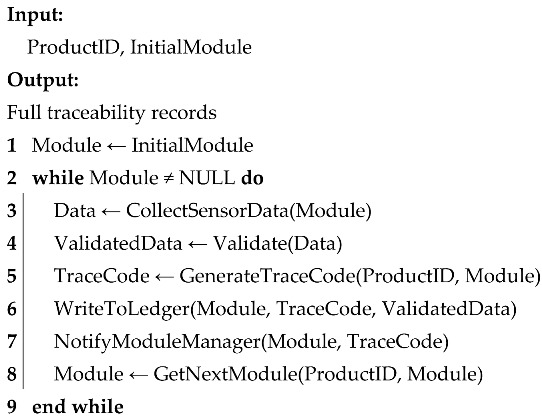


#### 3.2.2. Traceability Code Design

**Figure 6:**
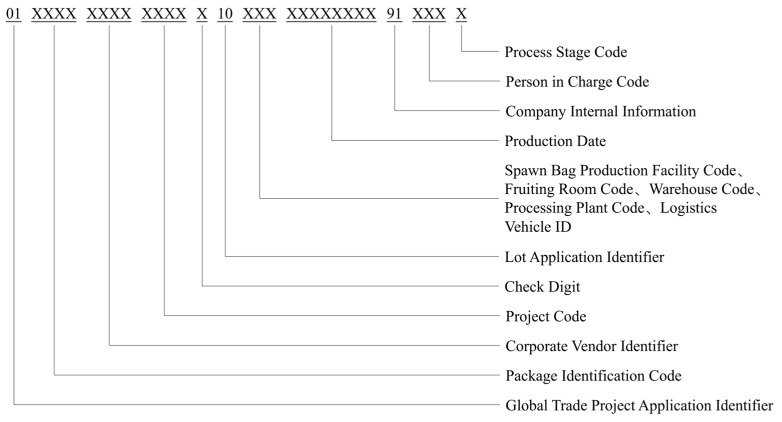
Traceability Code Structure for *Pleurotus ostreatus*.

To enable precise identification and information tracking throughout the entire oyster mushroom supply chain, this paper proposes an extended multi-field traceability code structure based on the GTIN-14 standard [43], as illustrated in Figure 6. The traceability code begins with the prefix “01,” complying with internationally recognized product coding conventions. It is further extended to accommodate practical business requirements, forming a flexible coding framework adaptable to multiple stages and cross-system applications.

The overall structure comprises multiple functional fields, including process code, operator code, enterprise identifier, batch number, timestamp, geographic location code, packaging identifier, and a check digit. Specifically, the process code distinguishes the business module to which the code belongs (e.g., inoculation, cultivation, storage); the operator code records responsible personnel to enhance traceability accountability; the enterprise/trademark identifier and packaging identifier define product ownership and packaging type; the batch number and timestamp ensure temporal consistency and batch uniqueness; and the location code enables precise positioning down to the park/workshop/warehouse level. In addition, a reusable application identifier is introduced to support unified traceability parsing across business scenarios, thereby improving the system’s interoperability and integration capabilities.

#### 3.2.3. Rapid Traceability Module

**Figure 7:**
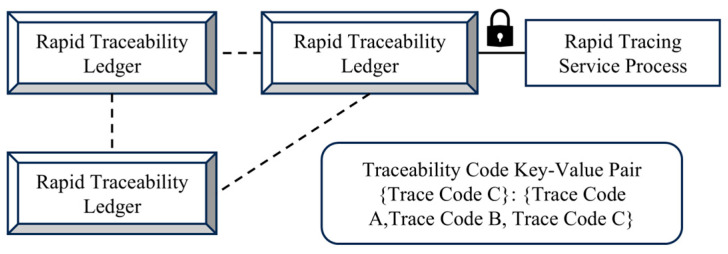
Architecture of the Rapid Traceability Module (The dashed lines represent the communication flow between the Rapid Traceability Ledger and the Rapid Traceability Service Process. The symbols represent encrypted traceability codes).

The rapid traceability module is the core component of traceability queries and consists of two parts, namely the rapid traceability service program and the rapid traceability ledger, as shown in Figure 7. This module plays a key role in enhancing query efficiency within the flexible traceability system by establishing an efficient link between data generation and traceability requests, ensuring system responsiveness and query completeness.

The rapid traceability service program acts as an intermediary between client commands and the ledger. It is primarily responsible for receiving and parsing user commands, and executing data write or query operations. When new product data are generated in any supply chain stage, the service automatically creates the corresponding traceability code and writes it along with key information to the ledger. Upon a user traceability request, the program quickly returns the complete traceability path based on the traceability code index. Moreover, the program integrates a data validity check mechanism to prevent abnormal or unauthorized data entries, thereby ensuring the accuracy and usability of ledger data. The procedure of traceability query execution is detailed in Algorithm 2.
**Algorithm 2**: Pseudocode for the Rapid Traceability Query Procedure.

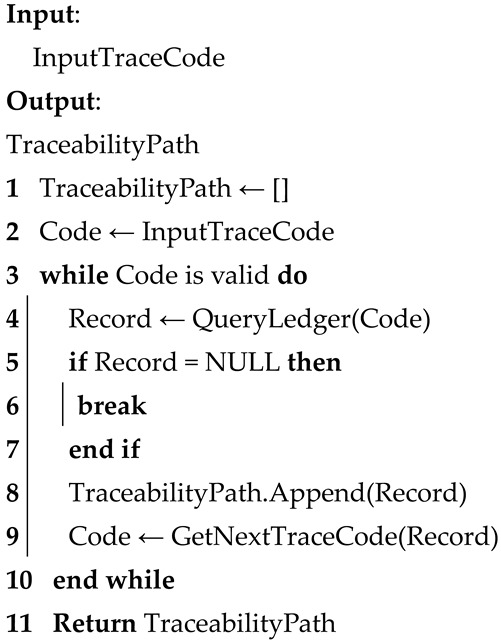


### 3.3. Multi-Entity Chain Storage Architecture

In the *Pleurotus ostreatus* supply chain, blockchain technology is used to establish public, private, and regulatory chains, enabling effective storage and sharing of information. Based on the differentiated needs of various traceability stakeholders, the traceability information of the *Pleurotus ostreatus* supply chain is classified as shown in Table 3.

To improve the efficiency and transparency of data queries, consumers using a public blockchain can quickly access plaintext data stored on the chain. Data on public blockchains is usually stored in plaintext form, allowing consumers to directly query relevant information through blockchain nodes, thereby enabling rapid retrieval of required information.

For enterprises, data security and privacy are more critical. Therefore, private blockchains employ the SHA-256 algorithm to protect traceability information by generating a 256-bit hash value through a compression function and uploading it onto the chain. Before storage, the traceability information is first converted into binary form and then encrypted into ciphertext using the RSA algorithm and stored in a local database. Additionally, Ciphertext-Policy Attribute-Based Encryption (CP-ABE) is applied to the ciphertext with an access policy vector set as the key. CP-ABE enables fine-grained control over data sharing, allowing data owners to flexibly define access policies based on user attributes. It demonstrates greater robustness and scalability in scenarios involving multi-user concurrency and dynamic permission adjustments. This capability is particularly critical for dynamic data sharing among multiple roles in the *Pleurotus ostreatus* supply chain [44,45,46]. Only when the attribute vector provided by the enterprise satisfies the access policy can the plaintext data be decrypted using the private key. This process ensures that information in the private blockchain is effectively protected while maintaining the privacy of sensitive enterprise data. Although CP-ABE introduces higher computational overhead, its flexibility and fine-grained access control in complex multi-role collaborative environments offer significant advantages over symmetric encryption and other traditional methods. This flexibility is particularly crucial in supply chains, where multi-party collaboration and dynamic permission management are essential. Traditional symmetric or public-key encryption methods may struggle to meet the need for fine-grained control and dynamic permission adjustments. The attribute-based nature of CP-ABE enables it to fulfill these requirements, making the trade-off in computational performance worthwhile [47,48].

Regulatory authorities require real-time supervision of data to ensure its accuracy and compliance. The InterPlanetary File System (IPFS) uses content addressing and encryption mechanisms, where a file’s unique identifier is the hash of its content; if the file content changes, the hash value also changes. To enhance data security, IPFS also supports a public-key encryption-based encrypted file system. Before uploading data to the IPFS network, the data is encrypted with a public key so that only authorized users holding the corresponding private key can decrypt and access the data.

**Figure 8:**
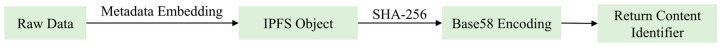
IPFS hash storage mechanism.

In the data management strategy of the regulatory blockchain, plaintext traceability information is uploaded to IPFS, and the hash value returned by IPFS is then uploaded to the blockchain. Blockchain nodes only back up these hash values and do not store the actual data. This not only significantly reduces data redundancy and storage pressure but also effectively ensures data integrity and security, as shown in Figure 8.

**Figure 9:**
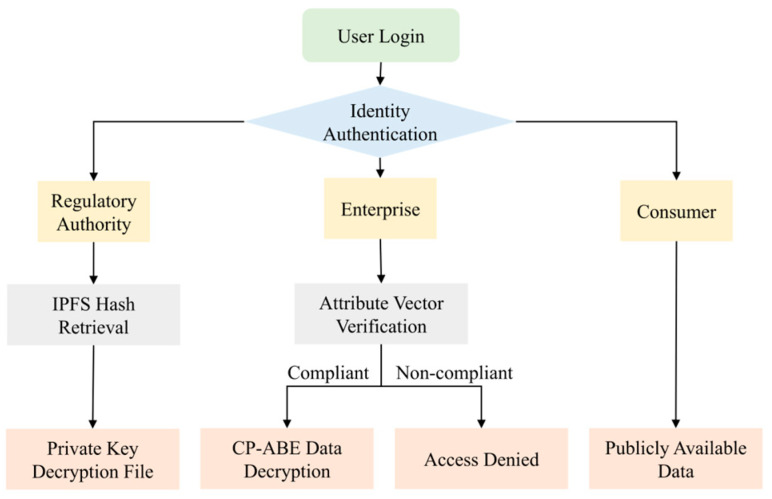
Multi-role access control mechanism.

Based on the differentiated requirements of various traceability entities, three levels of traceability data have been established according to identity and access permissions to ensure the privacy and security of information. This design strengthens enterprise data protection and improves cross-departmental collaboration efficiency. More importantly, it establishes a trustworthy, transparent, and traceable security framework for *Pleurotus ostreatus*, reducing contamination and spoilage risks while enhancing the overall level of food quality and safety. The multi-role chain storage structure of oyster mushroom is illustrated in Figure 9.

### 3.4. Traceability Information Acquisition System

Ensuring the stable and optimal conditions necessary for the growth of *Pleurotus ostreatus* requires the deployment of diverse sensors to achieve comprehensive multi-point monitoring. To improve the consistency and accuracy of multi-point data collection, the system employs a multi-sensor data fusion model based on an adaptive weighting algorithm. Suppose there are n similar sensors collect values as $x_{1},x_{2},\ldots ,x_{n}$, with corresponding variances $\sigma_{1}^{2},\sigma_{2}^{2},\ldots ,\sigma_{n}^{2}$. The optimal weights are calculated as follows:

(1) $$\omega_{i}=\frac{\frac{1}{\sigma_{i}^{2}}}{\frac{\sum_{j=1}^{n}\;1}{\sigma_{j}^{2}}}$$

where $\sigma_{i}^{2}$ is the historical measurement variance of the sensor of the ith one.

The fusion estimate is shown as follows:

(2) $$X=\sum_{i=1}^{n}\omega_{i}x_{i}$$

where the weights $\omega_{i}$ satisfy $\sum_{i=1}^{n}\omega_{i}=1$, $\omega_{i}\geq 0$.

To further improve robustness, an iterative optimization mechanism is introduced as follows.

1. Sort the measurements and calculate the extreme median value $Y_{0}$ as follows:
  (3) $$Y_{0}=\frac{Y_{min}+Y_{max}}{2}$$
2. Split the dataset according to $Y_{0}$, as shown in the following equation:
  (4) $$\left\{\begin{array}{c} Z_{1}=\{Y_{j}|Y_{j}<Y_{0}\}\\ Z_{2}=\{Y_{j}|Y_{j}\geq Y_{0}\} \end{array}\right.$$
  Calculate the subset weighted means ${E[Z}_{1}]$ and ${E[Z}_{2}]$.
  (5) $$E\left[Z_{1}\right]=\frac{\sum_{Y_{j}<Y_{0}}\;Y_{j}\times \omega_{j}}{\sum_{Y_{j}<Y_{0}}\;\omega_{j}}$$
  (6) $$E\left[Z_{2}\right]=\frac{\sum_{Y_{j}\geq Y_{0}}\;Y_{j}\times \omega_{j}}{\sum_{Y_{j}\geq Y_{0}}\;\omega_{j}}$$
3. Find the mean value of ${E[Z}_{q}]$ and ${E[Z}_{q-1}]$, update the median value and perform iteration; the specific formula is as follows:
  (7) $$Y_{q}=\frac{{E[Z}_{q}]+{E[Z}_{q-1}]}{2}$$
4. Further compare the values of $Y_{q}$ and $Y_{q-1}$; if they are not equal, repeat step three until the iterations are equal.
5. The optimal measurement inputs of the sensors in the mushroom room are finally obtained by the above steps, and the optimal weighting factor $\omega_{i}^{*}$ is recalculated to output the final fusion result $X^{*}$.

## 4. System Implementation

**Figure 10:**
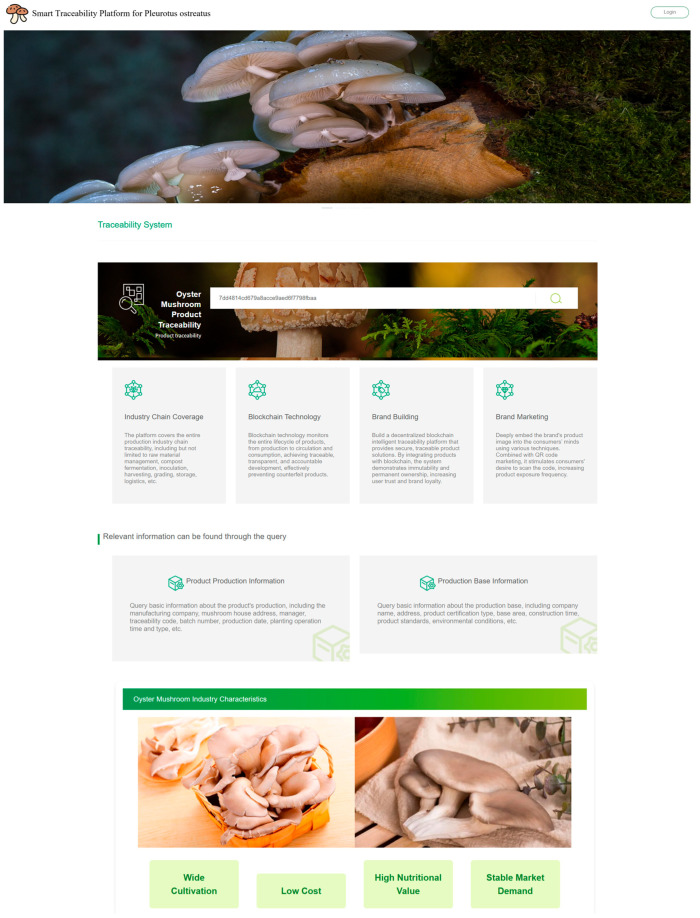
Homepage of the *Pleurotus ostreatus* Traceability System. (System entry interface for all users, featuring a dedicated “Oyster Mushroom Product Traceability” portal. Users can obtain product information by entering the traceability code or associated identifiers, with additional query channels such as “Production Base Information” and “Product Production Information.” The box shows the input field where the traceability code is entered, used for tracking the product’s history).

Based on the Hyperledger Fabric v1.4 framework, this study develops a multi-role blockchain-based flexible traceability system tailored for the oyster mushroom industry. The system homepage is illustrated in Figure 10. It is deployed in a cluster environment composed of four servers, comprising four Orderer nodes and two Peer nodes for each of the seven supply chain subchains. Kafka is adopted as the underlying consensus mechanism. The frontend integrates fabric-sdk-java to seamlessly bridge business logic with blockchain services and has been functionally adapted and optimized to meet actual business requirements.

This study was led by the Key Laboratory of Huang-Huai-Hai Smart Agricultural Technology, Ministry of Agriculture and Rural Affairs, which carried out all core tasks—system design and implementation, data acquisition, and analysis. The team comprised five members across the computer science, agricultural engineering, and food safety fields. This study was deployed at Shandong Qihe Eco-Agriculture Co., Ltd., involving 12 cultivation units and 60 LoRa sensor nodes. Data were collected from January to June 2024 across cultivation, storage, transportation, and sales. Environmental parameters were monitored using DS18B20 temperature sensors, SHT31 humidity sensors, and CO_2_ modules at 10-min intervals, while RFID tracked warehouse and logistics operations. Data were preprocessed via edge gateways and anchored on the Hyperledger Fabric blockchain, with raw logs stored off-chain and their hash indexes preserved on-chain for verification.

Challenges encountered during deployment included inconsistent sensor calibration, hardware performance variations, and integration with existing farm management systems. These were addressed through a unified calibration process and a middleware adaptation layer to ensure stable and reliable operation.

**Figure 11:**
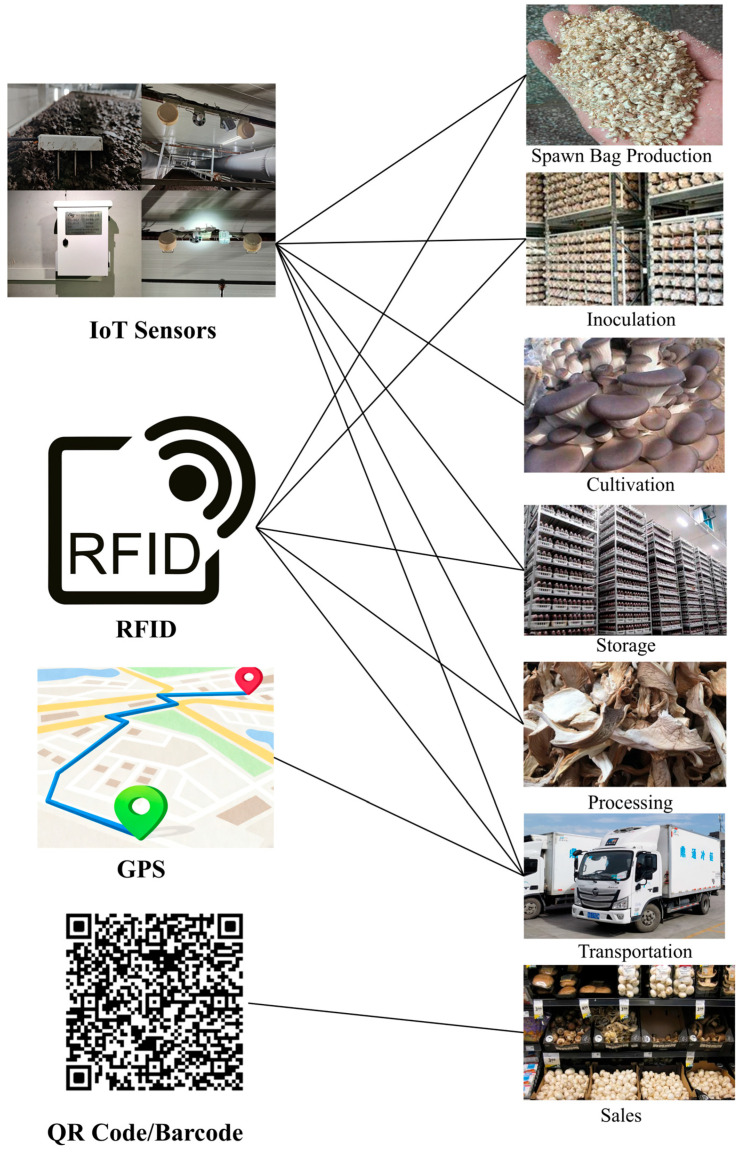
Traceability Information Collection Framework for *Pleurotus ostreatus*.

Based on this infrastructure, a full-process information collection framework was established. Substrate preparation focused on formulation and sterilization parameters; cultivation focused on temperature, humidity, and CO_2_ concentration; logistics focused on GPS and vehicle environment data to maintain cold-chain integrity; and sales focused on QR code-enabled consumer queries, as shown in Figure 11. By securely recording and tracing these key data, the system enables early detection of abnormal conditions, rapid localization of risks, efficient recalls, and reduced contamination or spoilage spread. This enhances quality and safety assurance for *Pleurotus ostreatus* and supports sustainable supply chain management.

**Figure 12:**
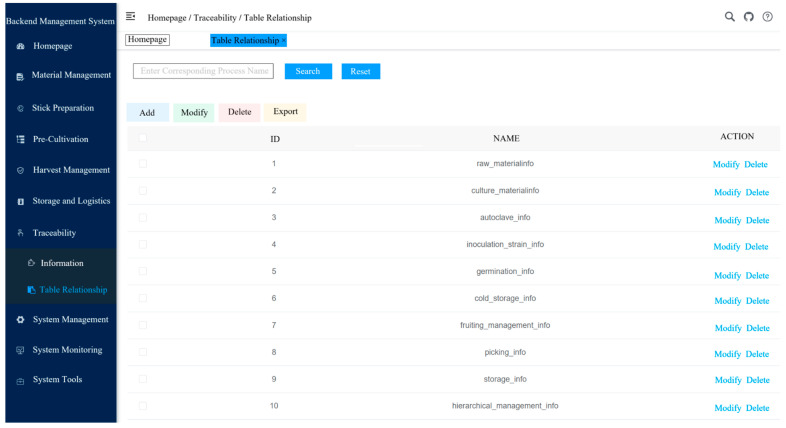
Diagram of the Backend Management System. (Core operational interface for enterprise administrators, highlighting “Comprehensive Production Process Management” and “System Monitoring.” The interface integrates modules such as raw material management, stick preparation and inoculation, pre-cultivation, and harvest management, with real-time alerts and control recommendations for dynamic risk prevention. It also incorporates portals for oyster mushroom nutritional data monitoring and system tools, supporting both quality traceability and enterprise-level system configuration. The meaning of the Chinese terms in the figure is as follows: The left side of the interface shows the main navigation menu, including modules like raw material management, culture material management, and harvesting management. The right side lists process flow data, such as “raw_materialinfo” (raw material info), “culture_materialinfo” (culture material info), and “fruiting_management_info” (fruiting management info), along with corresponding process numbers. Users can edit, delete, or export data by interacting with the icons).

**Figure 13:**
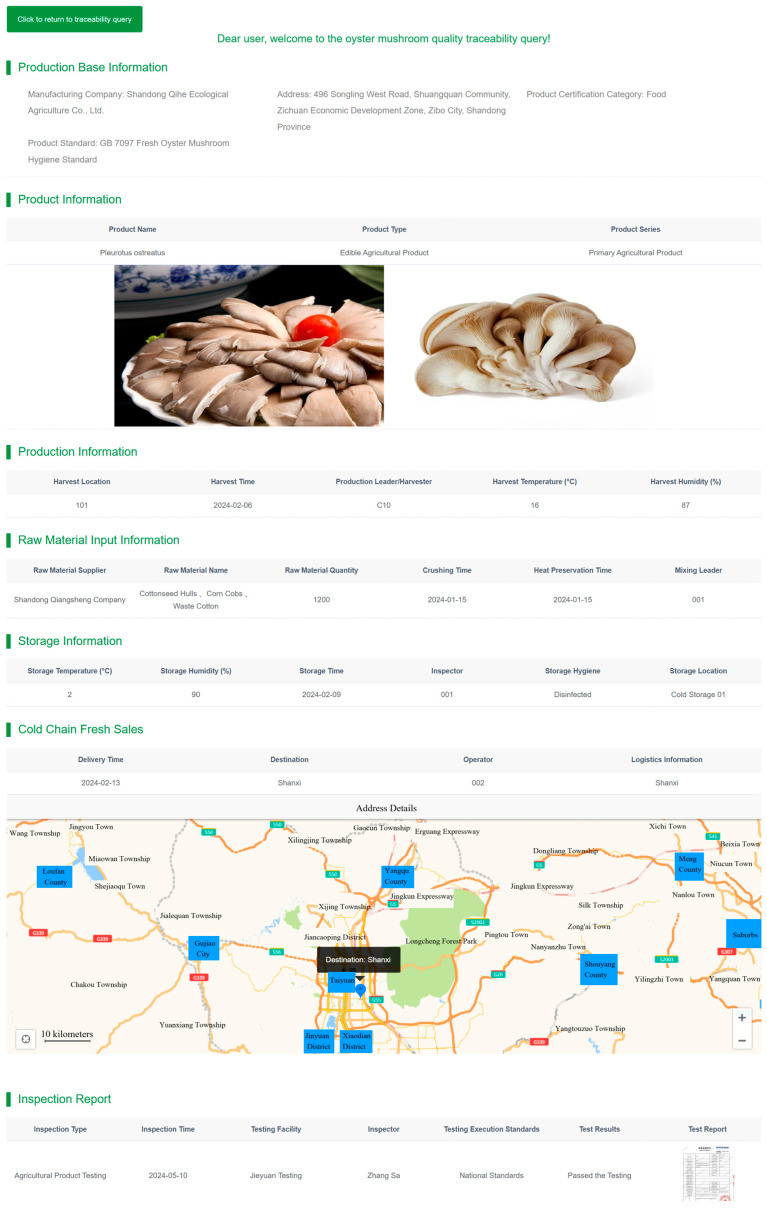
Visualization of Traceability Query Results. (Results display interface following a user-initiated traceability request. The interface presents key information across the entire oyster mushroom supply chain in a hierarchical manner, including environmental parameters (e.g., cultivation temperature and humidity), operational records (e.g., inoculation personnel, processing techniques), and quality inspection reports (e.g., heavy metal testing results)).

The system has remained in stable operation since deployment, accomplishing full-process traceability data collection and certification storage—from fungus stick production, inoculation, *Pleurotus ostreatus* cultivation, warehousing, processing, and transportation to sales (see backend management interface in Figure 12). To date, over 10,000 records have been stored on-chain, enabling role-specific queries for key production stage information by diverse stakeholders (sample query results shown in Figure 13).

Through a “stage–ledger” mapping approach, the system achieves segmented chain storage. Inter-chain coordination and scheduling are managed by the module management service, allowing flexible adjustment or recombination of traceability paths based on real-time production needs. Experimental results confirm that the proposed system significantly enhances the security and transparency of oyster mushroom traceability data and demonstrates clear advantages in trustworthiness and process flexibility compared to traditional systems.

## 5. Experimental Comparison

### 5.1. Experimental Validation of Data Collection

To evaluate the fusion mechanism’s effectiveness, we conducted an experimental comparison using air temperature as a representative parameter. Ten sets of temperature readings were collected from heterogeneous sensors and processed with three fusion methods: the arithmetic mean, the adaptive weighted fusion algorithm, and the improved adaptive weighted fusion algorithm proposed in this study. Table 4 presents the total variance of fusion results used for performance assessment. The results show that the improved algorithm significantly outperforms the others by effectively suppressing noise and compensating for sensor discrepancies. Compared to the arithmetic mean and the original adaptive algorithm, the improved version reduces total variance by 20%, demonstrating a clear improvement in fusion accuracy. Furthermore, a two-tailed *t*-test was conducted for significance analysis, and the results indicated that the improvement was statistically significant at the 95% confidence level (*p* < 0.05), confirming the robustness and effectiveness of the proposed method. This enhancement directly improves the accuracy and credibility of critical environmental traceability data, thereby strengthening the overall reliability of the system.

The improved adaptive weighted fusion algorithm not only enhances data accuracy but also significantly strengthens the system’s early warning capabilities. Through experimental comparisons, the system’s success rate in detecting real potential issues, such as temperature fluctuations and cold chain failures, increased by 15%. Specifically, the system detected 75 out of the 100 actual issues, compared to 60 detected with the original data. Among the 80 warnings issued by the system, the proportion of accurate warnings rose from 70% to 82%, resulting in a 12% improvement in precision.

The increase in early warning detection rate directly led to earlier identification of quality issues, reducing spoilage and losses caused by cold chain failures and environmental fluctuations. For example, during cold chain transportation, the system, based on fused data, identified a small temperature fluctuation that was not clearly apparent in the arithmetic mean or the adaptive weighted fusion algorithms. The system promptly issued a warning and took automated intervention measures, ensuring product safety and preventing the spoilage of hundreds of kilograms of flat mushrooms, thus ensuring food safety. By improving the adaptive weighted fusion algorithm, the system can better integrate signals from multiple sensors, enhancing both the accuracy and response speed of early warnings.

### 5.2. Advantages over the Single-Chain Model

In this section, the performance of the proposed model is analyzed experimentally. At present, data storage in the *Pleurotus ostreatus* supply chain is primarily based on a single-chain architecture. Here, a comparative analysis is conducted between the traditional single-chain storage approach and the proposed MFT-Chain model.

#### 5.2.1. Experimental Environment and Parameter Settings

To construct the blockchain test environment, a system was configured with 32 GB RAM, 1 TB hard drive, and 100 Mb/s network bandwidth. Hyperledger Fabric v1.4.2 was adopted as the blockchain framework, running on Ubuntu 16.04 with Docker 19.03 to ensure stable and efficient operation. The performance evaluation was conducted using the blockchain benchmarking tool Caliper.

#### 5.2.2. Data Query Efficiency Analysis

**Figure 14:**
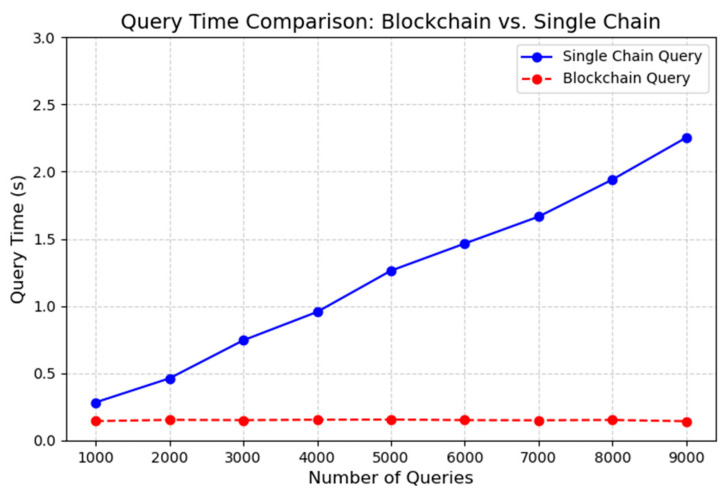
Comparison of Query Time Between Single-Chain and Block-Chain Models.

In terms of query performance, data volume was used as a variable to test query latency, and the results are shown in Figure 14. When performing traceability data queries, the query time of the single-chain model increases proportionally with the amount of data queried, whereas the query time of the MFT-Chain model remains relatively stable and consistently lower than that of the single-chain model.

#### 5.2.3. Throughput Analysis

**Figure 15:**
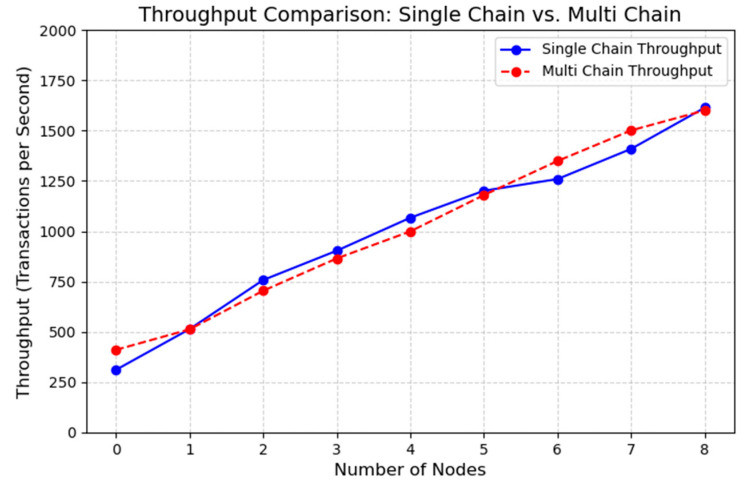
Comparison of Throughput Between Single-Chain and Block-Chain Models.

In terms of throughput, the number of nodes was used as a variable for testing. As the number of nodes increased, system throughput gradually improved. The results, shown in Figure 15, indicate that compared with the single-chain model, the MFT-Chain model is able to maintain higher throughput even under large-scale node settings, demonstrating its advantage in scalability.

#### 5.2.4. Resource Overhead Comparison

System energy consumption was measured using an external power meter with per-second sampling, while CPU utilization was recorded by the node monitoring program and summarized using P50/P95 statistics. The results show that, compared with the single-chain architecture, the MFT-Chain model reduces per-transaction energy consumption by approximately 12% and average CPU utilization by about 15%. Under the same throughput conditions, it also maintains lower latency fluctuations, indicating that the system achieves a more favorable balance between performance and resource utilization.

### 5.3. Verification of Pleurotus ostreatus Quality Monitoring and Spoilage Control Effectiveness

To verify the system’s practical effectiveness in food quality control, this study conducted a comparative monitoring of *Pleurotus ostreatus* storage and transportation quality before and after deployment at Shandong Qihe Ecological Agriculture Co., Ltd. A total of 60 sample batches, collected before and after system deployment, were compared in terms of spoilage rate, sensory quality scores, and quality inspection pass rates within 5 days post-harvest.

The monitoring results indicated that, after the system was implemented, the average spoilage rate of *Pleurotus ostreatus* batches decreased from 8.1% to 7.1% after 7 days, representing a reduction of 12.3%. The sensory quality score improved by approximately 0.8 points, and the quality inspection pass rate increased from 82.6% to 95.2%. Through real-time monitoring of temperature, humidity, and CO_2_ levels, the system was able to issue early warnings for abnormal batches. During two high-temperature fluctuation events, the system automatically triggered the regulation process, successfully preventing batch spoilage caused by temperature control failures.

Specifically, the improved adaptive weighted fusion algorithm enables the system to issue early warnings when potential quality issues are detected, resulting in a reduction in spoilage. With more precise data analysis, the system can promptly identify cold chain failures or contamination in the production process and issue alerts before the issues occur, thus preventing losses caused by delayed reactions. This enhanced data accuracy directly improves the responsiveness and precision of quality control.

These results demonstrate that the MFT-Chain system not only ensures data integrity and traceability through blockchain-based data storage but also significantly improves spoilage control and quality assurance during the storage and transportation of *Pleurotus ostreatus* through real-time monitoring and early warning mechanisms. This empirical evidence further confirms the applicability and feasibility of the proposed model in the field of food science.

## 6. Discussion

This study addresses the practical needs of the *Pleurotus ostreatus* supply chain by proposing a blockchain-based multi-chain flexible and trusted traceability model to improve the system’s performance in terms of data credibility, structural flexibility, and multi-stakeholder collaboration. Compared to the single-chain model, the MFT-Chain model effectively decouples business modules through a “link-chain” mapping mechanism, enhancing the system’s ability to dynamically reorganize. This provides a highly adaptive traceability solution for the complex and dynamic *Pleurotus ostreatus* supply chain. Experimental results show that the cross-chain query module significantly outperforms the single-chain storage model. In terms of throughput, as the number of nodes increases, the MFT-Chain model demonstrates good scalability, maintaining stable query efficiency and significantly outperforming the single-chain solution, making it suitable for large-scale agricultural products, especially high-risk foods like edible fungi, in safety traceability and emergency recall applications. Previous research has explored multi-chain or hybrid-chain traceability methods in agricultural and food sectors. For instance, Arvana et al. proposed a multi-chain architecture for food traceability in the meat value chain to enhance transparency and efficiency [49]. Additionally, a review on agricultural traceability highlighted challenges in the integration and widespread application of blockchain in agricultural traceability systems [50], including issues related to “scalability, interoperability, and integration with IoT.” Feasibility studies on the integration of agricultural Internet of Things and blockchain have empirically discussed issues such as low-power sensor devices, data transmission delays, and system overhead [51]. Moreover, the latest research on food quality traceability and dynamic monitoring has indicated that blockchain has seen initial applications in tracking the quality of fresh food and environmental monitoring [52]. Combining these findings, the MFT-Chain’s strategies in high-concurrency queries, cross-chain efficiency, and real-time quality monitoring integration offer significant competitive advantages over existing solutions.

From the perspective of food science, the system’s dynamic granularity control and real-time sensory data fusion not only improve the quality controllability of *Pleurotus ostreatus* during storage and transportation but also provide new empirical data to support research on the spoilage mechanisms of edible fungi. Through multi-dimensional monitoring of temperature, humidity, gas concentration, and mycelium quality indicators, MFT-Chain can digitally identify early signs of spoilage, triggering risk warnings in advance at the source and during circulation. This feature addresses the shortcomings of many blockchain-based food traceability studies that “only record, but do not diagnose,” laying the foundation for future food safety risk models based on intelligent sensing. Furthermore, through on-site verification, the system achieved a 12.3% reduction in spoilage rate and a 15.4% increase in quality inspection pass rate, demonstrating that it not only ensures data credibility and system scalability but also directly enhances food quality and safety levels in practical production environments.

Despite the successful validation of MFT-Chain’s feasibility in the *Pleurotus ostreatus* supply chain, there are still some limitations. First, the system depends on the stability of the LoRa network, and data loss or delays may occur in extreme environments. Second, the multi-chain architecture introduces additional overhead in node management and operation. Although the dynamic traceability granularity mechanism improves flexibility, it may face performance bottlenecks during large-scale concurrent queries. While the access control mechanism solves privacy leakage, the encryption based on Ciphertext-Policy Attribute-Based Encryption (CP-ABE) still has room for improvement in high-concurrency scenarios. Additionally, while the on-chain/off-chain collaborative storage and fast retrieval module improve storage and query efficiency, optimization is still needed in complex business scenarios.

Future research can focus on the following directions: first, introducing cross-chain interoperability and edge computing technologies to enhance data processing capacity and system stability under large-scale deployments, thereby improving real-time quality and safety support for food cold chain scenarios; second, exploring lightweight encryption schemes or zero-knowledge proof technologies to reduce the performance overhead of access control; third, combining hardware acceleration or strategy optimization to improve encryption and decryption efficiency; and fourth, using graph databases, cross-chain middleware, and multimodal data fusion methods to optimize query performance under complex conditions, meeting the needs of food regulatory agencies for fast verification of quality and safety information in complex supply chains.

## 7. Conclusions

This study, grounded in the practical needs of the *Pleurotus ostreatus* supply chain, proposes a blockchain-based multi-chain flexible and trustworthy traceability model. At the implementation level, the system integrates an architecture of “off-chain sensing and fusion + on-chain verification and certification.” An adaptive weighted fusion algorithm is employed to improve the quality of environmental data, while a fast retrieval module optimizes cross-chain data access efficiency. At the same time, mechanisms such as IPFS ensure a balance between data privacy and efficient storage.

Deployment tests conducted with *Pleurotus ostreatus* products demonstrate that integrating IoT with blockchain in mushroom traceability systems not only enhances data reliability and supply chain transparency but also provides new management tools for agricultural enterprises and regulatory authorities. For enterprises, the system enables rapid identification of quality issues, reduces recall costs, and strengthens brand credibility. For regulators, the multi-chain architecture and fine-grained access control support tiered supervision and accountability tracing. For consumers, the system increases trust in food safety, thereby promoting market acceptance and driving consumption upgrades.

The proposed flexible and trustworthy traceability model outperforms traditional schemes not only at the technical level but, more importantly, also shows significant application potential in quality control, spoilage risk prevention, and food safety supervision across the mushroom supply chain. In the future, it could be extended to multi-crop, multi-region, and cross-platform practices for agricultural product quality and safety traceability.

## Figures and Tables

**Table 1:** Analysis table of key issues in each link of the supply chain.

Process Stage	Quality Risk Issues
Fungus Stick Production	Heavy metal contamination in raw materials; Improper substrate formulation
Inoculation	Non-standardized inoculation; Incomplete inoculation records; Risk of cross-contamination
Cultivation	Contaminated fruiting environment; Heavy metal bioaccumulation risk
Processing	Poor sanitation; Cross-contact contamination; Non-compliant processing practices
Storage	Batch mixing; Expired or prolonged storage; Temperature and humidity deviations
Transportation	Temperature excursion; Delivery delay; Information gap
Sales	Label non-compliance; Missing origin information

**Table 2:** Traceability Granularity at Each Stage of the *Pleurotus ostreatus* Supply Chain.

Process Stage	Traceability Granularity	On-Chain Records	Off-Chain Storage
Fungus Stick Production	Raw material batch level	Supplier ID; Bagging event & timestamp; Operator ID; Equipment parameter hash	Supplier qualification docs; Bagging operation logs; Equipment parameter logs
Inoculation	Inoculation shift level	Inoculation event & timestamp; Strain source ID; Disinfection record hash; Sterilization parameter hash; Operator ID	Sterilization logs; Disinfection reports; Inoculation operation sheets
Cultivation	Fruiting shift level	Temperature/humidity/CO_2_ window hash; Heavy metal test summary & hash; Operator ID; QC report hash	Continuous sensor curves (CSV/Parquet); Heavy metal test reports (PDF); Field photos
Processing	Processing shift level	Key process parameter summary; Sterilization parameter hash; Process QC result summary; Operator ID; Compliance evidence hash	Equipment operation logs; HACCP/process forms; Packaging lists; QC reports (PDF)
Storage	Storage batch level	Inbound/outbound events; Warehouse location code; Temperature/humidity window hash; Abnormal event logs (e.g., temperature failure)	Cold storage temperature & humidity logs; WMS inbound/outbound records; Inventory reports
Transportation	Transportation batch level	Loading/unloading events & timestamps; Waybill number; Vehicle/carrier ID; Transport temperature hash; GPS path hash	Vehicle temperature curves; GPS trajectories; Electronic waybills; Carrier certificates
Sales	Individual item level	Public fields (batch, origin, production/packaging date, compliance summary, certificate/report hash); Privacy policy ID	Static disclosure page snapshots; Certificates (PDF, anonymized)

**Table 3:** Traceability information for each subject.

Process Stage	Consumers	Corporations	Regulatory Authorities
Production	Product grade, growth environment data, quality inspection records, harvest timestamp	Strain source (supplier, batch), inoculation time, operator, growth environment logs (temperature, humidity, light, CO_2_), operation logs, equipment operation status	Production license ID, phytosanitary certificate for spawn, raw material heavy metal test results, pesticide residue reports
Processing	Processing time, HACCP-compliant environment, quality attributes, inspection reports	Processed quantity, sterilization parameters, processing manager and operator, equipment operation logs, process settings (sterilization temperature, duration)	GMP certification, microbial test reports for raw and finished products
Storage	Storage temperature/humidity, storage duration	Inbound/outbound timestamps, inventory levels, warehouse operators, equipment status (cold storage monitoring, abnormal alarms, partition IDs and location codes)	Qualification documents, quality inspection reports, automated temperature records
Transportation	Real-time temperature during logistics, shipment milestones	Product classification, shipped quantity, logistics provider and vehicle ID, cold chain temperature records, en route alarm logs, arrival time, responsible personnel	Transportation license, temperature excursion reports, loading/unloading video records
Sales	Retailer ID, best-before date, POS data, organic certification	Traceability lot code, sales volume, sales channel (online/offline), return and complaint handling records	Sales environment details, qualification certificates, organic certification ID, point-of-sale temperature logs, label compliance audits, consumer complaint trace records

**Table 4:** Data fusion results.

Total Variance	Arithmetic Mean Method	Adaptive Weighted Fusion Algorithm	Improved Adaptive Weighted Fusion Algorithm
1	0.1548	0.0779	0.0681
2	0.1393	0.0625	0.0507
3	0.1449	0.0472	0.0371
4	0.1324	0.0593	0.0475
5	0.0908	0.0456	0.0361
6	0.0964	0.0507	0.0401
7	0.0895	0.0428	0.0332
8	0.0950	0.0494	0.0387
9	0.1242	0.0583	0.0461
10	0.1166	0.0489	0.0382

## Data Availability

Data can be made available upon request from the authors.

## Abbreviations

The following abbreviations are used in this manuscript:

CP-ABE	Ciphertext-Policy Attribute-Based Encryption
LoRa	Long Range Radio
IPFS	InterPlanetary File System
GTIN	Global Trade Item Number
IoT	Internet of Things
RFID	Radio Frequency Identification
GPS	Global Positioning System
PBFT	Practical Byzantine Fault Tolerance
P2P	Peer-To-Peer
